# Outcome for dizzy patients in a physiotherapy practice: an observational study

**DOI:** 10.1080/07853890.2022.2091790

**Published:** 2022-07-04

**Authors:** Willem De Hertogh, René Castien, Laura Jacxsens, Joke De Pauw, Luc Vereeck

**Affiliations:** a Department of Rehabilitation Sciences and Physiotherapy, Faculty of Medicine and Health Sciences, University of Antwerp, Antwerp, Belgium; b Primary Care Rehabilitation Practice Kineglazenleeuw, Beveren, Belgium; c Department of General Practice and Elderly Care Medicine, EMGO + Institute for Health and Care Research, VU University Medical Center, Amsterdam, The Netherlands; d Healthcare Center Haarlemmermeer, Hoofddorp, The Netherlands

**Keywords:** Dizziness, vertigo, therapeutic outcomes, therapy, primary care

## Abstract

**Background:**

Dizziness is a common reason for referral to physiotherapy. Additional information on clinical characteristics, treatment effect and prognostic indicators in physiotherapy practice are needed.

**Methods:**

A retrospective observational study. Based on a standardised clinical evaluation patients were labelled as having Benign Paroxysmal Positioning Vertigo (BPPV) or not (no-BPPV). BPPV was treated with repositioning manoeuvres and exercises. In no-BPPV, treatment was based on additional clinical tests. Treatment was provided once per week and considered successful when the patient was free of symptoms confirmed by negative positional tests.

**Results:**

From 148 referred patients, 88 were labelled as having BPPV, 60 as no-BPPV. The symptom of a short-lasting spinning sensation provoked by head movements was highly suggestive of BPPV. On average, in BPPV treatment was completed after 2.27 ± 1.68 treatments, in no-BPPV this was after 4.91 ± 3.46 treatments. The delayed outcome was related to higher ‘age’ and ‘concomitant neck pain’ in BPPV and with higher ‘age’ only in no-BPPV. Favourable outcome was related to the feature ‘dizziness provoked by movements in the horizontal plane’ in BPPV.

**Conclusions:**

Clinical evaluation and treatment in physiotherapy practice can be an effective and safe option for patients with dizziness. Several clinical variables with prognostic values were identified.

**Key messages**
1. Clinical evaluation and treatment in physiotherapy practice can be part of low threshold care for dizzy patients.
2. Despite prior medical screening, one-third of patients without signs of BPPV were sent back for further evaluation, illustrating the need for interdisciplinary collaboration.
3. Based on the description of the dizziness symptom (vertigo rather than light-headedness), provocation of the dizziness by movements, and a short duration of the dizziness attack, and positive clinical vestibular tests, BPPV treatment could be initiated.

## Introduction

Dizziness (including vertigo) is a common complaint. It affects about 15% to over 20% of adults yearly in large population-based studies [1]. The most common causes are cardiovascular and peripheral vestibular conditions [2]. The diagnosis is made based on thorough patient history and a clinical examination. Nevertheless, in a substantial part of patients, no specific cause can be determined [3]. In that sense, obtaining or just failing to obtain a therapeutic effect can assist the diagnostic process. Vestibular vertigo is believed to account for about a quarter of dizziness complaints and has a 12-month prevalence of 5% and an annual incidence of 1.4%. It is an important reason for patients to contact a physician such as a general practitioner (GP), neurologist or an Ear-Nose-Throat (ENT) specialist [4].

One of the peripheral otological causes of dizziness is Benign Paroxysmal Positional Vertigo (BPPV). BPPV is diagnosed on the basis of typical anamnestic characteristics such as short-term recurrent attacks of positional (spinning) dizziness of short duration accompanied by nystagmus [5]. When BPPV is suspected, an attempt is made to determine the type (canalo- or cupulolithiasis), affected side and affected canal. Specific clinical tests such as the Supine Roll Test and the Dix-Hallpike Test are commonly used for testing the horizontal and vertical (anterior and posterior) semicircular canals, respectively [6]. The posterior semicircular canal (PSCC) is most frequently affected, with a prevalence of up to 87% [7,8]. The horizontal canal (HSCC) is affected in 5–15% of patients [9]. BPPV can resolve spontaneously. In patients who visited the hospital within 3 days after the onset of their vertigo, the symptoms resolved within 7 days in 30% of PSCC and 58% of HSCC. Without treatment, vertigo lasted more than one month in 36% and 11% respectively [8]. For the treatment of PSCC-BPPV, the provision of advice and canalith repositioning manoeuvres such as the Epley manoeuvre are recommended [9]. When the horizontal canal is affected, the Barbeque Roll (Lempert) manoeuvre or the Gufoni/Appiani manoeuvre are recommended [9–13]. These repositioning manoeuvres, as part of vestibular rehabilitation (VR), are often performed by physicians and vestibular therapists [14] and are considered to be more effective than Brandt-Daroff exercises [10,15]. Data about success rates of VR for BPPV and about factors that might affect treatment outcomes in physiotherapy practice are rarely described [9,16,17]. However, this information seems essential and may guide clinical reasoning and treatment.

If dizziness becomes chronic, the initial cause of the dizziness is sometimes hard to determine. In these chronic patients, VR programs are prescribed in which trunk, head and neck movements that trigger the dizziness are performed repeatedly to stimulate central compensation and habituation. These VR programs are safe, effective and easy to administer [18,19].

Providing appropriate care in an easily accessible setting e.g. in primary care can avoid delaying the final diagnosis [20]. Information on prognostic indicators – clinical features that predict treatment outcome – can help to identify patients who may benefit or not from treatment. The early detection of patients with a high/low chance for treatment success can result in a more adequate referral of patients.

Consequently, we aimed to answer the following research questions:

1. What are the clinical characteristics of patients with dizziness who are referred to a primary care rehabilitation practice?
2. What is the effect of an individualised VR program on patients with and without BPPV in a primary care rehabilitation practice?
3. Which clinical characteristics are related to a favourable outcome of the individualised VR in patients with and without BPPV?

## Materials and methods

### Study design

A retrospective observational dossier study was carried out on patient files of consecutive patients who registered for dizziness between May 2016 and March 2020. We followed patients throughout their clinical flow and assessed their prognosis [21]. The study has been approved by the Ethical Committee of the Antwerp University Hospital (reference: 20/40/522) and was conducted according to the Declaration of Helsinki.

### Setting and participants

Files of patients who registered for dizziness in a primary care rehabilitation and physiotherapy clinic were considered. They were included when the patients were explicitly referred for dizziness by either a primary care physician or an ENT specialist. They contain clinical information from the start to the end of treatment. During each treatment session, a brief note was made about the patients’ progress and about the administered therapy. Files of patients with BPPV combined with other conditions were excluded from further analysis.

All patients were assessed and treated in a structured and standardised approach by the same investigator (WDH) to reduce the risk of measuring bias as much as possible. This investigator is a musculoskeletal physiotherapist with over 20 years of clinical expertise and a specific interest in complaints of the head and neck region, such as dizziness.

### Variables

Demographic variables such as gender and age were noted. The following dizziness variables were collected: description of symptoms, duration of the dizziness history and of the current episode, duration of an individual dizziness attack, provocative postures or movements, presence of accompanying symptoms such as headache, ringing in the ears/hearing loss, neck pain, neurovegetative signs such as nausea and palpitations, self-reported comorbidities and medication intake. All reported medications were checked in an online compendium [22], and the following subdivisions were made for the nature of the comorbidities: diabetes, cardiovascular (in case of medication for hypertension, hypercholesterolaemia, coagulation disorders, cardiac arrhythmias), ENT (when taking antiallergic medication or betahistine), neurological (in case of anti-epileptics) and other. Next, the positional tests Dix-Hallpike and/or supine roll test (see Supplementary Material, Appendix 1) were performed and eventual provocation of dizziness and the occurrence of nystagmus was noted.

As a measure of treatment success, the number of treatments required until the patient was free of symptoms was counted. The latter was defined as a self-reported absence of symptoms. In patients with BPPV, this outcome needed to be confirmed by negative positional tests. A clinical vestibular test was considered negative if no vertigo was provoked and no nystagmus was observed during testing. Visual fixation suppression might have been possible as Frenzel goggles or video-oculoscopy were not available.

### Measurements and procedures

#### Clinical investigation

All patients were systematically questioned according to a standardised document, collecting the items that are described above. Subsequently, all patients underwent a standardised clinical examination consisting of two vestibular clinical tests: the Dix-Hallpike manoeuvre for the posterior and anterior canals and, when the Dix-Hallpike manoeuvre was negative or provoked horizontal nystagmus, the Supine Roll Test for the horizontal canals [6,9].

Based on the anamnestic data, supplemented with the findings of the positional tests, all patients were labelled as having BPPV or another form of dizziness. In the group of BPPV the affected canal was noted as BPPV posterior canal left, right or both sides, or BPPV horizontal canal.

#### Therapy

All patients with BPPV were informed and counselled about their condition. This included an explanation of the anatomy and function of the semi-circular canals and the aetiology and prevalence of BPPV. Next, patients underwent a repositioning manoeuvre, targeting their affected canal.

In the case of PSCC-BPPV, the Epley manoeuvre was applied [9]; in the case of a BPPV of the horizontal canal, the Lempert manoeuvre was applied [23]. See Supplementary Material, Appendix 2 for a description. The repositioning manoeuvres were performed once during a single therapy session. If necessary, the manoeuvre was repeated in a subsequent weekly session which always started with a re-assessment.

In case of residual symptoms such as a discrete feeling of dizziness or unsteadiness, but negative positional tests, additional exercises as described by Lucy Yardley [24] or Cawthorne-Cooksey [9] were provided (see Supplementary Material, Appendix 3). This combination of repositioning manoeuvres and VR exercises is in line with Cochrane recommendations [25]. Treatment was ended when the patient reported being free of symptoms and when the positional tests were negative.

When no BPPV was present, patients were labelled as having another form of dizziness. They received physiotherapy based on the results of a non-vestibular assessment including clinical cervical and balance tests. In these patients exercises as described by Yardley [19] were administered. If patients experienced difficulties performing head movements due to neck stiffness or neck pain, they received cervical physiotherapy. Patients were referred back to the referring physician when no starting points for rehabilitation could be identified, when the further evaluation was considered necessary or when their condition did not alter as expected.

### Study size

Data were collected from patient files of consecutive patients referred by medical doctors. The sample can be regarded as a convenience sample, given its retrospective design and the setting where the study was carried out.

## Statistical analysis

To analyse the clinical characteristics of patients, descriptive statistics (frequencies and averages with standard deviations) were used. Differences between patients with and without BPPV were calculated *via* independent samples *t*-tests for continuous variables or Chi-Square for dichotomous variables.

To analyse the effect of individualised VR, the number of treatments needed to become symptom-free was counted.

To analyse which variables influenced therapy outcome we performed logistic regression analysis (backward stepwise) where the required number of treatments to obtain treatment success was dichotomised. For the BPPV group, the binary encoding as ‘requiring more than 3 treatments’ yes: 1/no: 0. In the non-BPPV group, this was ‘requiring more than 4 treatments’ yes: 1/no: 0. This classification was chosen based on the distribution of patients within both groups over the successive treatments. First, a univariable analysis was performed (significance level set at *p* < .20). Next, a multivariable analysis was performed including the significant variables from the univariable analysis (significance level set at *p* < .05). As a measure of the performance of the obtained models, the predicted probabilities were extracted to analyse a Receiver Operating Characteristics (ROC) Curve and the corresponding Area Under the Curve (AUC). Next, the Youden index, sensitivity and specificity were calculated.

## Results

In total, data from 148 patients were collected. Of these, 93 were female (63%) and 55 were male (37%). The mean age was 59.3 years ± 16.22 years. Patients were referred by an ENT specialist (80 patients, 54%) or general practitioner (65 patients, 44%). Only 3 patients presented without a referral (1.9%). The sample contained 88 patients with BPPV and 60 without BPPV. A description of the entire sample and for patients with and without BPPV separately is presented in Table 1.

**Table 1.** Description of the sample.

	ALL patients (*n* = 148)	BPPV (*n* = 88)	No-BPPV (*n* = 60)	*p*-value
Male/Female ratio	55/93	31/57	24/36	.49*
Age (years)	59.3 ± 16.22	58.7 ± 15.3	60.1 ± 17.5	.60†
Duration of complaints (weeks)	8.4 ± 19.8	5.3 ± 13.1	12.8 ± 26.1	.04†
Referral by ENT specialist	80	44	36	.49*
Referral by general practitioner	65	42	23	
Self-referral	3	2	1	

Comparisons of patients with and without BPPV (no-BPPV) are presented. †Independent samples *t*-test; *Chi-Square test.

### Clinical features of the entire sample

#### Symptom presentation

A spinning rotatory sensation was reported by 121 patients (81.8%). In nine patients this was combined with a feeling of light-headedness and in eleven patients with a feeling of imbalance. Twenty-four patients (16.2%) reported only a feeling of light-headedness, two patients had light-headedness combined with imbalance.

Provocation of the dizziness by movements or postures was reported by almost all patients. Only eight (5.4%) reported that their dizziness was not provoked by movements or postures. Provocation was felt when moving in the sagittal plane such as looking up or bending forward by 72 or 48.6% of patients, and in the horizontal plane such as looking over one's shoulder or turning around in bed by 76 or 51.4% of patients. Other frequently reported provocative activities were lying down in bed (43 patients or 29.1%) and standing up (21 patients or 13.6%). There was no difference in provocative movements or positions between patients with a PSCC or HSCC.

Accompanying symptoms are common. Nausea (52.7%), headache (40.5%), neck pain (41.2%) and tinnitus (21.6%) are most commonly reported. Multiple comorbidities are reported. Cardiovascular comorbidities are the most frequent (54 patients, 36.5%); ENT comorbidities are reported by 23 patients (15.5%); 8 patients report diabetes (5.2%); 16 report a history of falls (10.8%).

### Comparison of clinical features in patients with and without BPPV

A comparison of the symptom presentation, provocative movements, accompanying symptoms, duration of an attack and comorbidities was made. Although not statistically significant, the duration of complaints before contact seems shorter in patients with BPPV than in patients without BPPV (5.31 weeks ± 13.13 weeks versus 12.80 weeks ± 26.17 weeks; *p*: .07).

In the patients with BPPV, there was a unilateral posterior canal involvement in 82% of patients, the horizontal canal was affected in 15% of patients and the bilateral posterior canal was involved in 2.2%. No anterior semicircular canals were involved.

In Table 2, an overview of the statistically significant features is presented. Accompanying symptoms and comorbidities were equally present in patients with and without BPPV. An overview can be found in Table 3.

**Table 2.** Clinical features that are statistically different between patients with (*n* = 88) and without BPPV (*n* = 60).

	BPPV	No-BPPV	Chi-Square	*p*-value
**Dizziness description**				
Vertigo	92%	66.7%	15.41	<.001
Light-headedness	11.4%	41.7%	18.14	<.001
**Provocation by movements**				
In general	100%	86.7%	12.40	<.001
In horizontal plane	60.2%	38.3%	6.85	.009
Turning over in bed	43.2%	21.7%	7.31	.007
**Duration of attack**				
About one minute	97.6%	67.3%	23.93	<.001

Percentages represent the proportion of the group where the feature is present.

**Table 3.** comparison of accompanying symptoms and comorbidities between patients with (*n* = 88) and without BPPV (*n* = 60).

	BPPV	No-BPPV	Chi-Square	*p*-value
**Associated symptoms**				
Hearing loss or tinnitus	23.9%	18.3%	0.644	.422
Visual symptoms	5.7%	6.7%	0.061	.806
Neck pain	37.5%	46.7%	1.237	.266
Palpitations	11.4%	8.3%	0.360	.549
Nausea	46.6%	61.7%	3.253	.071
Headache	34.1%	50.0%	3.746	.053
**Comorbidities**				
Diabetes	5.7%	5%	0.032	.857
Cardiovascular	34.1%	40%	0.538	.463
Ear-nose-throat	15.9%	15%	0.022	.881
Non-steroidal anti-inflammatory drugs	5.7%	1.7%	1.479	.224
Neurological	3.4%	0%	2.088	.148
Other	9.1%	16.7%	1.917	.166

Percentages represent the proportion of the group where the feature is present.

### Evaluation of treatment success in BPPV

**Figure 1.**
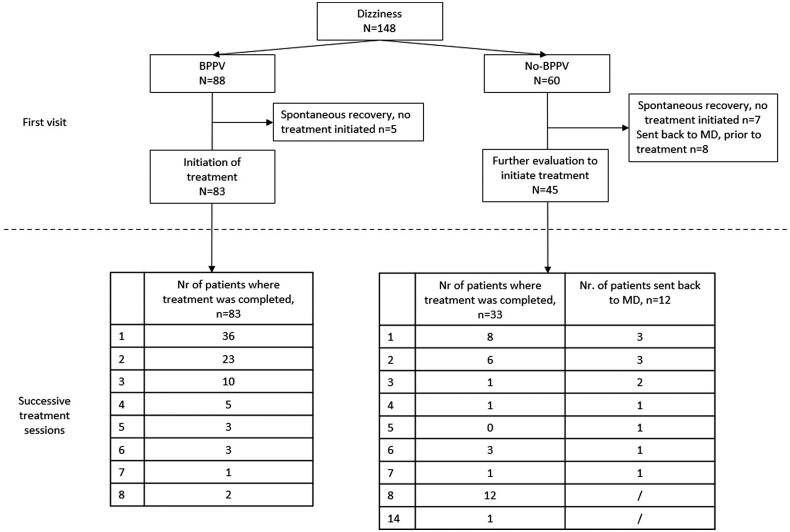
The patient flow of the entire sample per category (BPPV and No-BPPV). An overview of the number of required treatments is given.

In our sample, 88 patients were considered as BPPV only. In 5 patients, the first patient contact consisted only of an assessment and no treatment was considered necessary. In 83 treated patients, the number of required treatments until the patient was free of symptoms was counted (combination of the self-reported absence of symptoms and negative positional tests). This served as a measure of treatment success. On average 2.26 ± 1.68 treatments were required. An overview of the number of required treatments is presented in Figure 1.

### Evaluation of treatment success in patients without BPPV

In total, 60 patients were labelled as having no BPPV. At the first visit, spontaneous symptom resolution had occurred in 7 patients, and 8 were referred back to the physician for additional investigations. In 45 patients treatment was started. On average, 4.91 ± 3.46 treatments were needed (significantly different from the BPPV group, *p* < .0001). An overview of the number of required treatments is presented in Figure 1.

All patients who were referred back to the referring doctor were asked to provide feedback on the final diagnosis. Not all responded, but we were able to receive the following answers from four patients: hyperventilation, herpes zoster, temporal arteritis, residual complaints of commotio cerebri. Overall, 20 patients (i.e. 33.3%) were sent back to the referring medical doctor for further evaluation.

### Factors influencing therapy outcome

In Tables 4 and 5, the prognostic indicators for therapy outcome in BPPV and non-BPPV are displayed, respectively. In BPPV, variables that predicted the need for more than 3 treatments, are age and concomitant neck pain. Provocation of the dizziness by head movements in the horizontal plane predicts the need for 3 or fewer treatments (see Table 4). The AUC of the predictive model is 0.79, with a sensitivity of 0.79 and specificity of 0.66. In patients without BPPV, only age predicted the need for more than 4 treatments (See Table 5). The AUC of the predictive model was 0.69, with a sensitivity of 0.86 and a specificity of 0.48.

**Table 4.** Prognostic indicators for treated BPPV patients (*n* = 83) requiring more than 3 treatments.

	Univariable analysis			Multivariable analysis		
Variable	OR	CI	*p*	OR	CI	*P*
Age (years)	1.04	1.00–1.07	.04	1.05	1.01–1.10	.015
Provocation by looking up	0.35	0.09–1.34	.13			
Provocation by turning around in bed	0.40	0.14–1.10	.08			
Provocation by movements in horizontal plane	0.43	0.17–1.14	.09	0.25	0.07–0.81	.021
Neck pain	1.95	0.74–1.95	.195	4.42	1.31–14.91	.017
History of falls	3.73	0.77–18.16	.10			
Cardiovascular comorbidity	2.69	1.00–7.20	.05			
Neurological comorbidity	5.27	0.46–61.11	.18			
Comorbidity, other	2.75	0.63–12.05	.180			

**Table 5.** Prognostic indicators for requiring more than 4 sessions in non-BPPV.

	Univariate analysis			Multivariate analysis		
Variable	OR	CI	*p*	OR	CI	*P*
Age (years)	1.04	1.01–1.08	.03	1.07	1.01–1.12	.015
Duration complaints (weeks)	1.02	0.99–1.05	.18			
Nausea	0.35	0.10–1.23	.10			
Headache	0.44	0.13–1.45	.18			

## Discussion

In this retrospective cohort study, we evaluated the clinical characteristics of patients with dizziness who were referred by their general practitioner or ENT specialist for vestibular rehabilitation to a primary care physiotherapy practice. Additionally, we evaluated the patient's treatment outcomes and looked for prognostic factors for treatment success.

Patients were labelled as having BPPV or not using a standardised approach based on a combination of history taking and positional tests. Our analysis showed that from the history taking the description of the dizziness symptom (vertigo versus light-headedness), provocation of the dizziness by movements, and a short duration of the dizziness attack were highly suggestive of BPPV [26]. This is in line with the Barany Society's recommendations where this combination is considered to correspond with an episodic vestibular syndrome, such as BPPV [27,28]. The majority of patients reported a spinning sensation which can be regarded as vertigo. However, not all patients with this symptom pattern were designated as BPPV patients. It is known that patients experience difficulty in consistently describing their symptoms [29], and symptom description alone is not sufficient to guide diagnostics [26]. Hence, positional tests need to be conducted as well. They are not time-consuming and easy to perform. Based on these positional tests, we found that the posterior canal was most commonly affected in patients with BPPV, the horizontal canal was less affected. This is in line with reported percentages in the literature of 80–90% for the posterior canal [30]. Frenzel goggles or video-oculoscopy were not available in this physiotherapy setting. So, during the positional tests, they were not used. Nevertheless, we were able to rate the test result based on the provocation of the dizziness and the observation of nystagmus. To facilitate the observation of nystagmus, positional tests were performed with the head turned 45° away from the examiner. In the supine position, the patient then looks away from the examiner, making it less easy for the patient to focus visually on e.g. buttons or patterns on the examiner's clothing. By looking at the overlying eye and slightly pulling the eyelid sideways, the examiner can observe nystagmus. When visual suppression was suspected, a white paper was held before the patient’s eyes at a short distance to homogenise the visual field (Ganzfeld technique) [31].

As accompanying symptoms of their dizziness, patients reported primarily nausea, but also head and neck pain. These symptoms were only taken into account if they were more present during the episode of dizziness than in the period before. The concomitant neck pain can be a secondary symptom when patients keep their heads still to avoid provoking the dizziness in a head-on-trunk stiffness reaction [32].

Although not statistically significant, patients with BPPV tended to have a shorter duration of their complaints in comparison with patients without BPPV (5.3 versus 12.8 weeks, respectively). This can indicate that patients with BPPV gained relatively quick access to therapy, probably due to the awareness among referrers that repositioning manoeuvres are effective in this patient group. In the subjects where the diagnosis of BPPV was excluded, the referral process was slower and perhaps spontaneous dynamic compensation was awaited (until chronic vertigo (>3 m)).

Patients were referred by ENT specialists and general practitioners. Only a few patients arrived *via* self-referral. In Belgium, general practitioners are the gatekeepers of the health care system. They are often the first contact of the dizzy patient. General practitioners rule out underlying pathologies that might require specific attention [2]. ENT specialists focus on diagnosing vestibular causes. In the current sample, there was no difference in the prevalence of BPPV in patients referred by the GP or by the ENT specialist. Medical doctors' prior screening can explain why other causes of dizziness, such as dizziness caused by medication, were less present in our sample. Despite this prior medical screening, one-third of patients without BPPV were sent back to the referring doctor for further evaluation. This illustrates the complexity of dizziness and the need for interdisciplinary collaboration.

Treatment for patients with BPPV consisted of a combination of canalith repositioning manoeuvre (primarily Epley) and habituation exercises. In the early phases of treatment, the focus lies on the repositioning manoeuvre. Exercises were added after a few treatments when the vertiginous sensations subsided. In literature, this combination of repositioning with habituation exercises was found to be more effective than repositioning alone [33] and is indicated for patients with persistent symptoms after repositioning manoeuvres or with non-specific dizziness [9].

Not all patients required treatment: spontaneous recovery occurred in 5 patients with BPPV, and no VR was started (5%). This seems a lower percentage than the recovery rates that can be found in the literature. For instance, Imai *et al.* report a spontaneous recovery rate of 30% in patients with BPPV within a week after the onset [8]. The mean duration of complaints at the first visit in our sample was five weeks. Spontaneous recovery will probably have taken place in these first weeks. Our results indicate that spontaneous recovery can still occur after a few weeks, and it can also contribute to the clinical improvement that was recorded in the following weeks. Data on the evolution per week are scarcely reported. Our reported number of treatments corresponds with the number of weeks before the dizziness resolved. In the elderly, about 70% improved within 7 weeks with a combination of repositioning manoeuvres and VR [34]. This information on the course of BPPV when treated can be useful: when the expected result of VR is delayed or absent, additional steps in the diagnostic process can be taken, for instance, referral for additional investigations. In this way, rehabilitation and physiotherapy can contribute to the diagnostic process.

No control group or randomisation was used in the current study, so no conclusions on treatment effect can be made. However, we believe the observed clinical outcome is in line with existing evidence on VR and illustrates the potential and importance of physiotherapy as initial care for dizzy patients.

Treatment in non-BPPV patients was based on additional clinical tests, consequently more individualised and more diverse. They received exercises as described by Yardley [19] and those who experienced difficulties performing head movements due to neck stiffness or neck pain, received cervical physiotherapy. A higher number of treatments was needed to reach the point of sufficient recovery. Our health care system allows the standard prescription of 9 rehabilitation treatments. This can explain why 12 of 33 treated patients actually use the maximum number of prescribed treatments.

We were able to identify several prognostic indicators. Higher age corresponds with a higher number of required treatments in patients with and without BPPV. The odds ratios seem low (1.05 and 1.07, respectively). However, this means that per year, the baseline odds increase multiplicatively (baseline odds X OR^nr of years^). This multiplicative nature indicates that increasing age is a factor to be taken into account when evaluating a patient's prognosis. This is in line with a systematic review that showed that older adults with BPPV require more repositioning manoeuvres to obtain treatment success [35]. In patients with BPPV, the prognostic model contained also ‘concomitant neck pain’ with an OR of 4.42 and ‘provocation of the dizziness by movements in the horizontal plane’ with an OR of 0.25. Having concomitant neck pain is associated with higher odds to need additional treatments. In older patients, neck mobility is often restricted [36] which can affect the performance of the positional tests and the repositioning manoeuvres. Neck pain or reduced neck mobility can result in a less extended position of the head during the repositioning manoeuvre, which can hamper the effectiveness of the manoeuvre. Therefore we believe it is important to instruct primary care clinicians about neck-friendly manoeuvres. In the current study, the repositioning manoeuvres were performed with extra care in elderly with fragile necks, for instance by sustaining the neck with both hands, and no adverse events were noted. Other less arduous techniques have been described such as the Gans and the Galleti-Contrino manoeuvre [37,38].

The provocation of the dizziness by movements in the horizontal plane (OR 0.25) was associated with a lower number of required treatments. This can be explained by the similarity of movements in the horizontal plane with the last phase of the Epley and Gans manoeuvre. It certainly encourages clinicians to perform repositioning manoeuvres in patients who report this type of provocation.

In primary care, general practitioners are sometimes reluctant to perform repositioning manoeuvres because they are little familiar with it [39]. An interdisciplinary collaboration e.g. with a skilled physiotherapist is in these cases recommended.

Our sample contained almost twice as many females as males. This higher prevalence in females is reported in the literature [1]. The overall median age of 60 years reflects the increasing prevalence of dizziness with age. The general population is ageing and people continue to live longer at home. This will lead to an increased presence of people with dizziness in primary care settings. Therefore, awareness and education on dizziness for primary care clinicians, including physiotherapists, is needed [14].

In our sample, BPPV was the most prevalent form of dizziness which is in line with the literature [1,40]. Given this high prevalence was found in a primary care physiotherapy practice, this implies that physiotherapists need to be aware of this form of dizziness, and should be able to assess and treat patients.

## Conclusion

We used a standardised approach consisting of a combination of history taking and positional tests to assess and treat dizzy patients. This approach can be easily applied in primary care. We were able to identify patients with and without BPPV and start treatment accordingly. This primary care approach can offer a form of low threshold care for dizzy patients.

## Data Availability

The data that support the findings of this study are openly available in Zenodo at http://doi.org/10.5281/zenodo.5873693, reference number 5873693.
